# RIOK3 keeps MDA5 inactive

**DOI:** 10.18632/oncotarget.5265

**Published:** 2015-08-26

**Authors:** Ken Takashima, Hiroyuki Oshiumi, Tsukasa Seya

**Affiliations:** Department of Microbiology and Immunology, Graduate School of Medicine, Hokkaido University, Sapporo, Japan

**Keywords:** MDA5 phosphorylation, RNA sensing, RIO kinase, type I interferon, autoimmune disorder

RIG-I and MDA5 are cytoplasmic RNA sensors that recognize dsRNA patterns and activate MAVS to induce innate antiviral gene program. RNA sensing is regulated by ubiquitination in RIG-I while by phosphorylation in MDA5. Thus, *de novo* phosphorylation is an essential step for keeping MDA5 inactive. We identified a protein kinase RIO kinase 3 (RIOK3) targeting MDA5, and here discuss its possible relationship to autoimmune diseases.

RNA pattern-sensing is a pivotal event in host defense against virus infections, which induces innate immune response, inflammation, and augmentation of lymphocyte functions. These are rooted in RNA sensor-mediated dendritic cell (DC) maturation. TLR3, 7 and 8 in endosome and RIG-I and MDA5 RNA helicases in cytoplasm are involved in RNA sensing in DCs. MDA5 recognizes relatively long double-stranded RNA yielded as a virus replication intermediate, leading to the formation of MDA5 filament required for activating the adaptor MAVS, then inducing IRF3 activation followed by type I IFN production. [1] Poliovirus, EMCV and measles virus are representative virus species recognized by MDA5.

**Figure 1:**
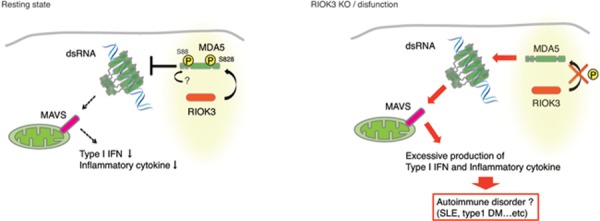
Sensing dsRNA by MDA5 MDA5 is oligomerized in the recognition of dsRNA, which leads to activation of the MAVS pathway. RIOK3 promotes S828 phosphorylation by which MDA5 is inactivated disabling multimer formation (left panel). Once RIOK3 is disrupted, MDA5 potentiates MAVS activation to produce excessive inflammatory mediators (right panel), which may cause autoimmune disorders.

Notably, only a little RIG-I and MDA5 exist in resting cells and viral infection markedly up-regulates their mRNA levels in affected cells. Then, the proteins are activated sufficient to recognize cytoplasmic RNA. For RIG-I activation, ubiquitin ligases TRIM25 and Riplet are indispensable, whereas no ligase is responsible for MDA5 activation. Recent report suggested that MDA5 was activated by dephosphorylation by PP1 [2]; if so, phosphorylation of *de novo* MDA5 is a prerequisite for keeping MDA5 inactive. We identified that RIO kinase 3 (RIOK3) phosphorylates MDA5 to be inactivated (Figure 1). RIOK3 selectively promotes C-terminal Ser- 828 phosphorylation of MDA5, which blocks MDA5 multimerazation and attenuates MDA5 signaling. [3]. Although another kinase might phosphorylate N-terminal region of MDA5, phosphorylation brings a dysfunctional conformation to MDA5 [2].

Excess activation of MDA5 was recently reported to link the process of autoimmune diseases such as SLE and type I diabetes [4]. Viral infections sometimes trigger autoimmune disorders as reported clinically [5]. However, the mechanism by which autoimmune diseases are exacerbated by MDA5 over-activation remains undetermined. G821S mutation near the Ser- 828 in MDA5 appears to be associated with constitutive activation of MDA5 and closely links to autoimmune triggering [6]. In virus infections or oncogenesis, RNA is released outside the affected cells with exosomes. Thus, the source of MDA5 ligands would be provided through RNA replication or cell destruction. However, how RIOK3 is regulated in cells that take RNA into the cytoplasm is unknown yet. RIOK3 knockout (RIOK3 KO) in culture cells produced more robust type I IFN and inflammatory cytokines than wild-type cells in response to polyI:C or viral infections, which can increase MDA5 levels (Takashima K et al, unpublished data). The results infer that RIOK3 KO surely promotes activation of MDA5 (Figure 1).

We are aware that the autoimmune disorder involves a number of signal axes in a variety of cells in patients. Regulatory T cells (Treg), B cells producing anti-DNA/RNA Ab, and other lymphocytes are involved in the process of autoimmunity. We notice here that innate immune response to RNA may trigger autoimmune disorder. Recent reports further suggest that Regnase-1 vs Loquin recognize 3′-stem-structured mRNA and regulate cytokine production such as IL-6 and TNF−α, which may suppress autoimmune disorder [7]. RIG-I and MDA5 are upstream of the cytokine producing gene program. Regulatory mechanism of RIG-I mechanistically differs from that of MDA5. What happens for MDA5 activation in oncogenesis, which contrasts to autoimmunity, is also intriguing. What is the role of RIOK3 in the pathogenic process of autoimmunity in the context of MDA5 activation will be the next issue to be analyzed. Since MDA5 is ubiquitously expressed, what cell types are responsible for a trigger of auto-reactive lymphocytes is a coming topic. We find we are in a new gate to the clue for the mechanistic mystery of RNA-dependent induction of autoimmune diseases.

We have elucidated the process of MDA5 activation, by which it recognizes cytoplasmic RNA. Excess RNA production via viral infections or tumor growth allows the cells to liberate a large amounts of structured RNA and facilitate autoimmune disorders (Figure 1). We may find a new strategy to the early diagnosis, prevention and treatment of autoimmune diseases by investigating RIOK3 knockout mice.
